# Auditory and Vestibular Characteristics of NLRP3 Inflammasome Related Autoinflammatory Disorders: Monogenic Hearing Loss Can Be Improved by Anti-interleukin-1 Therapy

**DOI:** 10.3389/fneur.2022.865763

**Published:** 2022-04-29

**Authors:** Hiroshi Nakanishi, Satoshi Yamada, Junya Kita, Daichi Shinmura, Kumiko Hosokawa, Sosuke Sahara, Kiyoshi Misawa

**Affiliations:** ^1^Department of Otorhinolaryngology/Head and Neck Surgery, Hamamatsu University School of Medicine, Hamamatsu, Japan; ^2^Department of Otorhinolaryngology, Numazu City Hospital, Numazu, Japan

**Keywords:** NLRP3, inflammasome, cryopyrin-associated periodic syndrome, hearing loss, DFNA34, anakinra, canakinumab

## Abstract

Inflammasomes are large multimeric protein complexes which regulate the activation of the proinflammatory cytokines interleukins-1β and−18 and inflammatory cell death called pyroptosis. NLRP1, NLRP3, NLRC4, AIM2, and pyrin can induce the formation of inflammasomes. Of these, the NLRP3 inflammasome is the most well-characterized. Recent studies revealed that variants of the *NLRP3* gene cause genetic diseases, including systemic inflammatory syndrome called cryopyrin-associated periodic syndrome (CAPS) and non-syndromic sensorineural hearing loss DFNA34. *NLRP3* variants cause CAPS and DFNA34 by constitutively activating the NLRP3 inflammasome and increasing IL-1β release. Patients with CAPS show systemic inflammatory symptoms, and hearing loss is a characteristic feature. Patients with CAPS and DFNA34 show progressive bilateral sensorineural hearing loss. Hearing loss has unique characteristics that can be improved or stabilized by anti-interluekin-1 therapy, although it is usually difficult to alleviate genetic hearing loss by drugs. However, it should be noted that there is a window of opportunity to respond to treatment, and younger patients are most likely to respond. It is important to know the characteristics of CAPS and DFNA34 for early diagnosis, and mutation analysis of *NLRP3* will lead to a definite diagnosis. In this review, we summarize the current understanding of the mechanisms of the NLRP3 inflammasome and characteristics of patients with CAPS and DFNA34, especially focused on auditory and vestibular findings.

## Introduction

Inflammasomes are large multimeric protein complexes known for their ability to regulate the activation of the proteolytic enzyme caspase-1 (1). Caspase-1 regulates the maturation of interleukins-1β and−18 (IL-1β and IL-18) through the proteolytic cleavage of pro-IL-1β and pro-IL-18, as well as an inflammatory cell death called pyroptosis (2). Assembly of inflammasome complexes is dependent on the cytosolic sensing of pathogen-associated molecular patterns (PAMPs) or danger-associated molecular patterns (DAMPs). PAMPs can access the cytosol and activate the innate immune system during microbial infections. DAMPs are endogenous danger signals released from dying or damaged cells and activate inflammasomes during non-infectious inflammation (3).

Sensors of PAMPs and DAMPs in the cytosol include nucleotide-binding oligomerization domain and leucin-rich-repeat-containing receptors (NLRs), absent in melanoma-2 (AIM2)-like receptors (ALRs), and proteins that contain a tripartite motif, such as pyrin. Among these sensor proteins, some NLRs including NLRP1 (NLR family pyrin domain containing 1), NLRP3, and NLRC4 (NLR family caspase-activation and recruitment domain-containing protein 4), together with AIM2 and pyrin, can induce the formation of inflammasomes (4). Inflammasome activation is critical for eliminating microbial infections and repairing damaged tissues. Conversely, the aberrant activation of inflammasomes is associated with inflammatory disorders, infectious diseases, and cancers. Studies in the past decade have shed light on the importance of appropriate activation of the inflammasome in homeostasis and disease pathogenesis (4).

Of these inflammasomes, the NLRP3 inflammasome is the most well-characterized. Recent studies revealed that variants of the *NLRP3* gene cause genetic diseases, including cryopyrin-associated periodic syndrome (CAPS) and non-syndromic sensorineural hearing loss (DFNA34, deafness autosomal dominant 34) (5, 6). The NLRP3 inflammasome is also involved in the initiation or progression of diverse disorders, such as obesity, type 2 diabetes, atherosclerosis, heart disease, inflammatory bowel diseases, gut microbiome, rheumatoid arthritis, Parkinson's disease, Alzheimer's disease, multiple sclerosis, and amyotrophic lateral sclerosis (7). In these disorders, sensorineural hearing loss was found in subjects with CAPS and DFNA34. Hearing loss has unique characteristics that can be improved by anti-interleukin-1 therapy, although it is usually difficult to improve genetic hearing loss by drugs (6). Therefore, it is important to know the characteristics of CAPS and DFNA34 for the early diagnosis and treatment. The available review articles on CAPS do not comprehensively summarize the auditory and vestibular findings, including clinical characteristics, imaging findings, response to anti-interleukin-1 therapy, and auditory performance after cochlear implantation (CI). In this review, we summarize the current understanding of the mechanisms of the NLRP3 inflammasome and characteristics of patients with CAPS and DFNA34, especially focused on auditory and vestibular findings.

## Methods

We obtained relevant literature published between 2012 and 2021, from PubMed and Embase, using the following medical terms: NLRP3, inflammasome for the section of NLRP3 inflammasome; CAPS, NOMID (neonatal-onset multisystem inflammatory disease), MWS (Muckle–Wells syndrome), FCAS (familial cold autoinflammatory syndrome), hearing loss for the section of CAPS; DFNA34 for the section of DFNA34. The inclusion criteria were defined as papers from clinical series or reviews published in relevant journals with rigorous materials and methods. The exclusion criteria were defined as papers not available in full-text or papers not written in the English. Additional papers published before 2012 were identified through the references in the published literature. Full-text articles written in English language were obtained.

## NLRP3 Inflammasome

**Figure 1:**
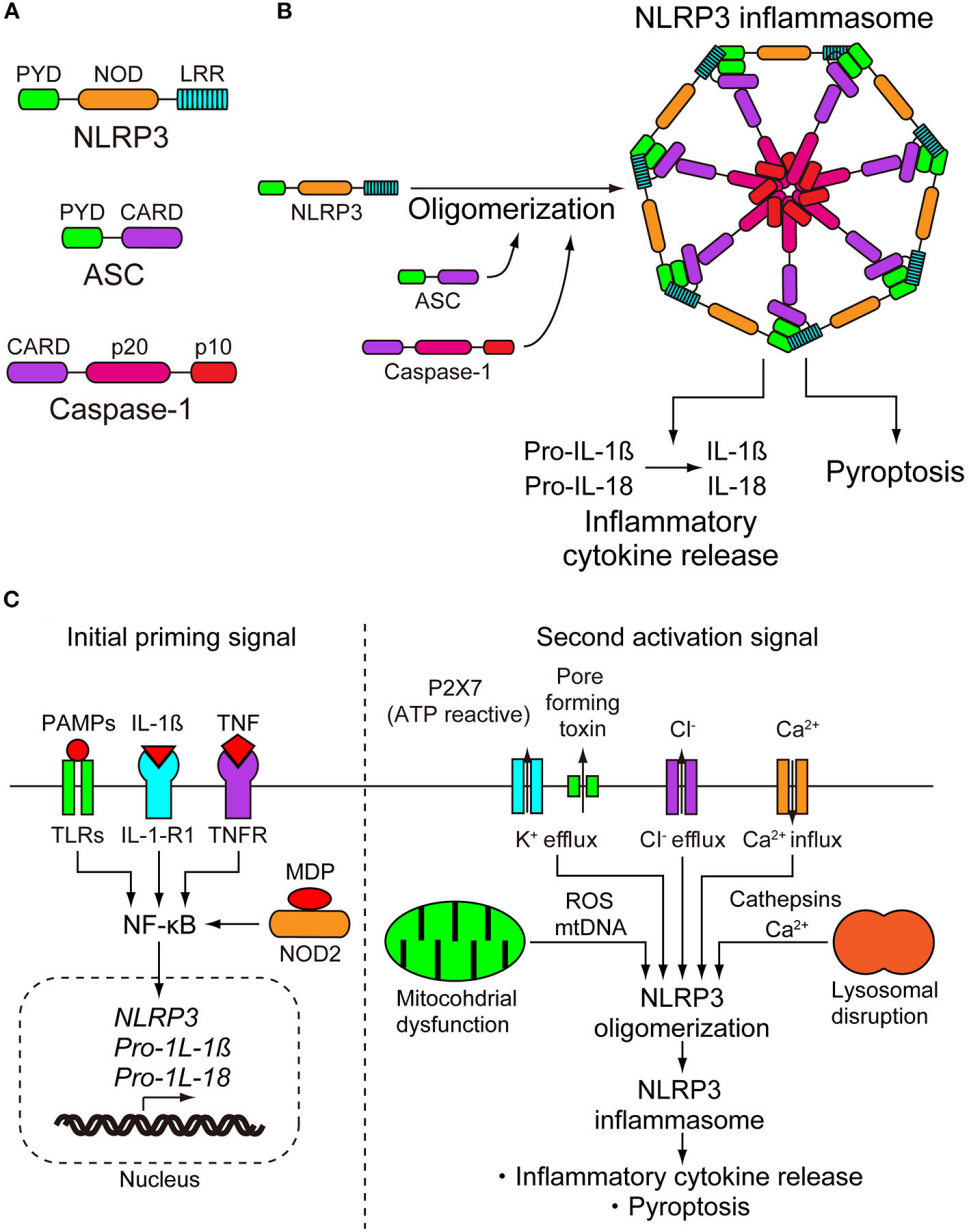
NLRP3 inflammasome. **(A)** NLRP3 inflammasome consists of NLRP3, apoptosis-associated speck-like protein containing a caspase-recruitment domain (ASC), and caspase-1. NLRP3 consists of an N-terminal pyrin (PYD) domain, a central nucleotide-binding oligomerization domain (NOD), and a leucine-rich repeat (LRR) domain at the C terminus. ASC has two interaction domains, N-terminal PYD and C-terminal caspase-recruitment domain (CARD). Caspase-1 has N-terminal CARD, a central large catalytic domain (p20), and C-terminal small catalytic domain (p10). **(B)** On stimulation, NLRP3 oligomerizes through the homotypic interactions of NODs. Oligomerized NLRP3 recruits ASC through PYD-PYD interactions and induces filament formation of ASC. Multiple ASC filaments combine to form a macromolecule. Assembled ASC recruits caspase-1 through CARD-CARD interactions, leading to formation of the NLRP3 inflammasome, and enables self-cleavage of caspase-1. Caspase-1 assembled on ASC self-cleaves into a complex of CARD-p20 and p10. CARD-p20 is proteolytically active and induces the maturation of interleukins-1β and−18 (IL-1β and IL-18) and pyroptosis. **(C)** Activation of NLRP3 requires a two step-signals, priming and activation. The initial priming signal causes the upregulation of the expression of NLRP3 inflammasome components NLRP3, pro-IL-1β, and pro-IL-18. The transcriptional upregulation can be induced through the recognition of pathogen-associated molecular patterns (PAMPs) and damage-associated molecular patterns (DAMPs). PAMPs and DAMPs are recognized by pattern recognition receptors including toll-like receptors (TRLs) and nucleotide-binding oligomerization domain-containing protein 2 (NOD2) or by cytokines including IL-1β and tumor necrosis factor (TNF), leading to nuclear factor-κB (NF-κB) activation and gene transcription. The second activation step begins with the recognition of NLRP3 activator and induction of NLRP3 inflammasome formation. NLRP3 can be activated by a wide range of unrelated stimuli. Due to their biochemical diversity, it is suspected that they induce a common cellular pathway. However, multiple signaling events have been observed in the activation of NLRP3 inflammasome, including efflux of potassium or chloride ions, flux of calcium ions, disruption of lysosomes, dysfunction of mitochondria, and production of reactive oxygen species. To date, there is no consensus model for the activation of the NLRP3 inflammasome. IL-1-R1, IL-1 receptor type 1; MDP, muramyl dipeptide; ROS, reactive oxygen species.

The NLRP3 inflammasome consists of a sensor protein NLRP3, an adaptor protein known as apoptosis-associated speck-like protein containing a caspase-recruitment domain, and an effector protein caspase-1 (Figure 1A) (8). NLRP3 is a tripartite protein that consists of an N-terminal pyrin (PYD) domain, a central nucleotide-binding oligomerization domain (NOD), followed by a leucine-rich repeat (LRR) domain at the C-terminus (9). NOD has ATPase activity that is required for NLRP3 oligomerization and function (10). ASCs have two interaction domains: an N-terminal PYD and a C-terminal caspase-recruitment domain (CARD). Full-length caspase-1 has an N-terminal CARD, a central large catalytic domain (p20), and a C-terminal small catalytic domain (p10) (11). Upon stimulation, NLRP3 oligomerizes through the homotypic interaction of NODs (Figure 1B) (10). Oligomerized NLRP3 recruits ASC through PYD–PYD interactions and induces filament formation with ASCs. Multiple ASC filaments combine to form a macromolecule, which is known as an ASC speck (12, 13). The assembled ASC recruits caspase-1 through CARD–CARD interactions, leading to formation of the NLRP3 inflammasome, and enables self-cleavage of caspase-1. Caspase-1 assembled on ASC self-cleaves into a complex of CARD-p20 and p10. CARD-p20 is proteolytically active and induces the maturation of IL-1β and IL-18 and the inflammatory cell death called pyroptosis (14).

Because activation of the NLRP3 inflammasome is directly linked to the inflammatory process, it must be tightly regulated. With only a few exceptions, the activation of NLRP3 is a two-step signaling process. In the first step, it is primed, and then it is activated. Initial priming signals have at least two functions, the first of which is to upregulate the expression of the NLRP3 inflammasome components NLRP3, pro-IL-1β, and pro-IL-18. The second function is to induce post-translational modifications of NLRP3, which changes the auto-suppressed state into a non-suppressed state (8). Transcriptional upregulation can be induced through the recognition of PAMPs and DAMPs. PAMPs and DAMPs are recognized by pattern recognition receptors, including toll-like receptors (TRLs) and nucleotide-binding oligomerization domain-containing protein 2 (NOD2), or by cytokines including IL-1β and tumor necrosis factor (TNF), leading to nuclear factor-κB activation and gene transcription (Figure 1C) (15–17). Post-translational modifications of NLRP3 include ubiquitylation and phosphorylation. Some of them work by activating regulatory modifications, while others work as negative regulatory modifications (18).

After the initial priming step, the second step begins with the recognition of the NLRP3 activator and induction of the NLRP3 inflammasome formation. Although pattern recognition receptors are specific for one or more related PAMPs or DAMPS, NLRP3 can be activated by a wide range of unrelated stimuli (19). The NLRP3 inflammasome can be activated in bacterial, fungal, and viral infections, as well as in inflammation caused by endogenous DAMPs and environmental DAMPs. Endogenous DAMPs include ATP, cholesterol crystals, urate crystals, amyloid-β, heme, and oxidized mitochondrial DNA. Environmental DAMPs include alum, silica, aluminum, and nanoparticles (8). Due to their biochemical diversity, it is suspected that they induce a common cellular pathway. However, multiple signaling events have been observed in the activation of the NLRP3 inflammasome, including efflux of potassium or chloride ions, flux of calcium ions, disruption of lysosomes, dysfunction of mitochondria, and production of reactive oxygen species (Figure 1C). Although there is abundant data describing these multiple signaling events, the pathways are interrelated and overlapping, and the data are conflicting in some cases. Therefore, there is no consensus model for the activation of the NLRP3 inflammasome (18). The details of these signaling events are summarized in the review articles described by Swanson et al. and Rathinam and Fitzgerald (8, 20).

## CAPS

### Overview of CAPS

CAPS is a spectrum of autosomal-dominant systemic autoinflammatory diseases caused by gain-of-function variants in the *NLRP3* gene (5). CAPS includes three clinical subtypes: neonatal-onset multisystem inflammatory disease (NOMID, also known as chronic infantile neurological cutaneous and articular syndrome), Muckle–Wells syndrome (MWS), and familial cold autoinflammatory syndrome (FCAS). These subtypes share common symptoms, including urticaria-like rash, recurrent fever, fatigue, arthralgia/myalgia, conjunctivitis/keratitis, and headache, with differences in the severity and length of inflammatory episodes (Table 1) (21).

**Table 1:** Clinical features of patients with CAPS (NOMID, MWS, and FCAS), and of those with non-syndromic sensorineural hearing loss DFNA34.

	**NOMID**	**MWS**	**FCAS**	**DFNA34^**a**^**
Cutaneous	Urticaria-like rash	Urticaria-like rash	Urticaria-like rash	
Systemic	Fever/fatigue	Fever/fatigue	Fever/fatigue	
Musculoskeletal	Arthralgia/myalgia/ bone deformity	Arthralgia/myalgia	Arthralgia/myalgia	
Ocular	Conjunctivitis/keratitis/ papillitis	Conjunctivitis/ keratitis	Conjunctivitis/ keratitis	
Auditory	Hearing loss	Hearing loss		Hearing loss
Central nervous system	Chronic aseptic meningitis	Headache	Headache	
Episode pattern	Chronic with 1–3 day flare	1–3 days	1–2 days	
Triggers	Stress/exercise/infection	Stress/exercise/infection	Generalized cold	

*CAPS, cryopyrin-associated periodic syndrome; NOMID, neonatal-onset multisystem inflammatory disease; MWS, Muckle-Wells syndrome; FCAS, familial cold autoinflammatory syndrome*.

a *Patients with DFNA34 show hearing loss without any other target-organ manifestations of CAPS*.

NOMID, the most severe form of CAPS, usually occurs at birth or early childhood and is characterized by intermittent fever, urticarial rash, and persistently elevated acute-phase reactant levels with typical facial features such as frontal bossing and saddle back nose. The central nervous system (CNS) manifestations include chronic aseptic meningitis, that can lead to brain atrophy and severe intellectual disability, if untreated. Chronic polyarthritis, leading to bone deformity, is also typical (22). FCAS, the mildest form of CAPS, is characterized by recurrent episodes of rash, fever, arthralgia, and conjunctivitis that are triggered by cold exposure. Most people have a normal life span, and developmental complications are rare (23). The intermittent form of MWS is characterized by recurrent attacks of rash, fever, arthralgia, and conjunctivitis, which are typically not triggered by cold exposure. Patients with MWS develop more severe ocular and CNS symptoms than patients with FCAS, including uveitis, episcleritis, and headache (24). Hearing loss is characteristic of NOMID and MWS, but is rarely found in FCAS (22, 24).

**Figure 2:**
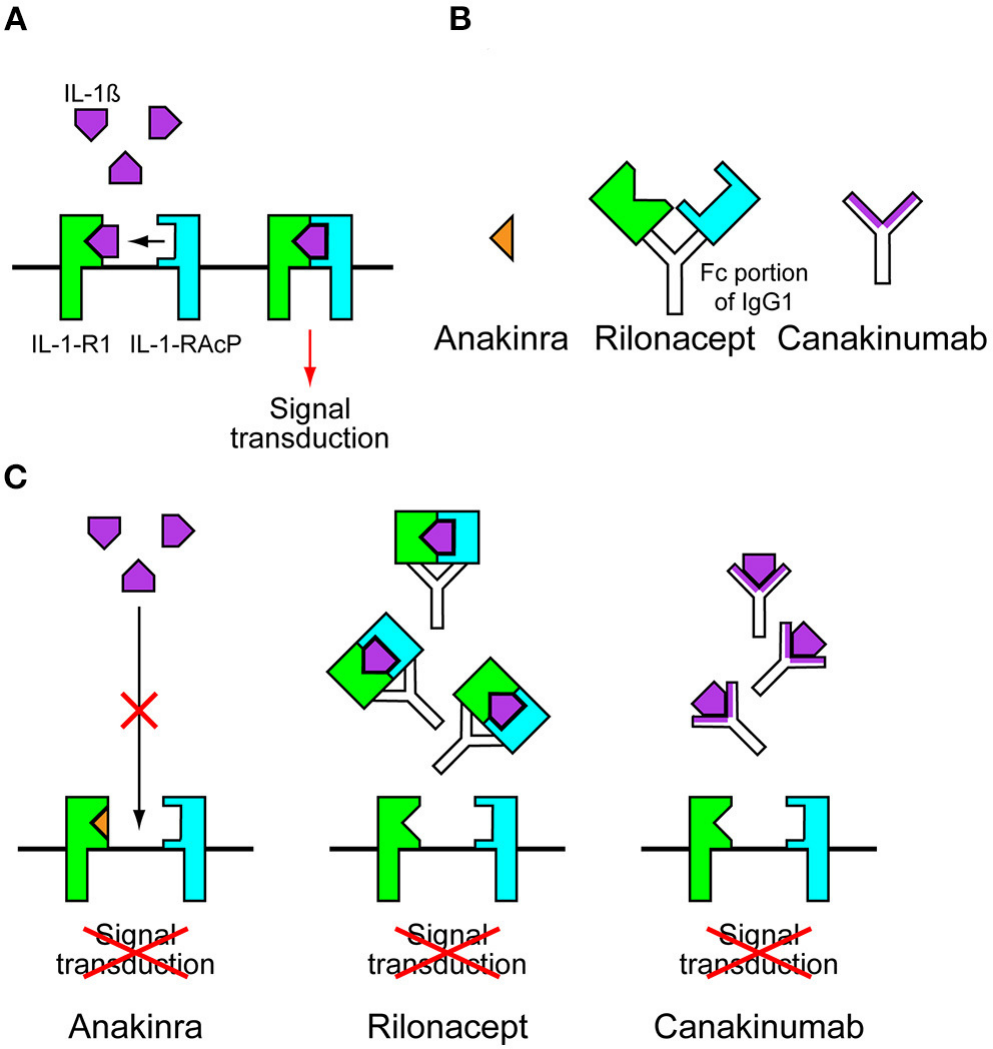
Anti-interleukin-1 drugs. **(A)** Interleukins-1β (IL-1β) activates cells by binding and signaling through IL-1 receptor type 1 (IL-1-R1) and the IL-1 receptor accessory protein (IL-1-RAcP). These receptors form a complex with IL-1β at the cell membrane for signal transduction. **(B)** Anakinra is a recombinant form of IL-1-R1 antagonist. Rilonacept is a dimeric IL-1 receptor fusion protein consisting of the extracellular domains of the IL-1-RAcP and the IL-1-R1 fused to the fragment crystallizable (Fc) portion of human immunoglobulin G1 (IgG1). Canakinumab is a specific human monoclonal IgG1 antibody against IL-1β. **(C)** In the presence of anakinra, IL-1β cannot bind to IL-1-R1. In the presence of rilonacept or canakinumab, Il-1β is trapped before it can reach its receptors, preventing signal transduction.

*NLRP3* variants cause CAPS by leading to the constitutive activation of the NLRP3 inflammasome and increased IL-1β release. This is supported by *in vitro* studies using monocytes extracted from patients with CAPS. Monocytes from patients with CAPS only require an initial priming signal to induce IL-1β secretion, whereas wild-type control cells require a second activating signal (25). IL-1β activates cells by binding and signaling through IL-1 receptor type 1 (IL-1-R1) and the IL-1 receptor accessory protein (IL-1-RAcP). These receptors form a complex with IL-1β at the cell membrane for signal transduction (Figure 2A) (26). Anakinra, a recombinant form of IL-1-R1 antagonist, prevented all CAPS symptoms in patients with NOMID, MWS, and FCAS (Figures 2B,C) (27–29). The daily use of anakinra was later approved by the Food and Drug Administration (FDA) and the European Medicine Agency (EMA). The success of anakinra in CAPS suggested that inhibiting IL-1-mediated inflammation was sufficient to prevent symptoms in patients with CAPS, and this was supported by a similar clinical success with additional IL-1-targeted drugs, such as rilonacept and canakinumab. Rilonacept is a dimeric IL-1 receptor fusion protein consisting of the extracellular domains of the IL-1-RAcP and the IL-1-R1 fused to the fragment crystallizable (Fc) portion of human immunoglobulin G1 (IgG1) (Figure 2B). It binds IL-1β with a high affinity and inhibits IL-1β activity (Figure 2C). Canakinumab is a specific human monoclonal IgG1 antibody against IL-1β (Figures 2B,C) (26). Rilonacept and canakinumab have a longer half-life than anakinra. Weekly dosing of rilonacept or every 2 months of canakinumab provided a similar efficacy in patients with MWS and FCAS in clinical trials (30, 31). These successes led to the FDA approval of using rilonacept in 2008 and canakinumab in 2009 for patients with CAPS. Currently, new drugs with likely applications for patients with CAPS are under development. These new drugs include small molecule inhibitor called MCC950 and ketone metabolite β-hydroxybutyrate (BHB). MCC950 is a potent and specific inhibitor of NLRP3 and blocks NLRP3 activation by directly targeting NODs of NLRP3, thus preventing NLRP3 oligomerization (32, 33). BHB inhibits the activation of NLRP3 inflammasome by preventing efflux of potassium and reducing ASC oligomerization and speck formation (34).

To date, 115 pathogenic/likely pathogenic *NLRP3* variants have been reported in patients with CAPS on infevers database (https://infevers.umai-montpellier.fr/web; accessed on 12/19/2021), and most of them affect the NOD domain of the NLRP3 protein. Some variants affect other regions of the protein, including the LRR domain. There are a few variants that cause one phenotype in some families and different phenotypes in other families. However, variants identified in patients with FCAS were not found in patients with NOMID. Thus, there is a fairly consistent genotype-phenotype correlation along the disease continuum (21). Somatic mosaicism for *NLRP3* variants has been observed in some patients with NOMID without significant CNS inflammation and in a few patients with MWS/FCAS. Patients with somatic mosaicism often show atypical phenotype with a late-onset (35, 36). In these cases, variants cannot be identified by standard Sanger sequencing because a small percentage of myeloid lineage cells have mutant alleles (37). In addition, low penetrance variants have been reported in some patients with atypical CAPS and in unaffected people with no significant signs or symptoms. In these cases, *in vitro* functional examinations using their monocytes were less functional than those in patients with typical CAPS (38).

### Auditory Characteristics of Patients With CAPS

Auditory characteristics in patients with MWS have been described in several reports, but reports of those of patients with NOMID are rare, probably due to difficulties in evaluating exact auditory function in patients with brain atrophy and intellectual disability.

Patients with MWS usually show a bilateral progressive sensorineural hearing loss. In most cases, the hearing loss is symmetric. At the onset, initially high frequencies are affected, and then the thresholds in the middle and low frequencies deteriorate. The hearing loss progressively worsens with age and can lead to a profound sensorineural hearing loss. Thus, the rate of patients with MWS affected by hearing loss increases with age (39, 40). Annual threshold deterioration reportedly ranged from 1.3 to 1.9 dB/year with the highest value at the lower frequencies among six affected members of a Dutch family with MWS, although progression rates are different among families with different *NLRP3* variants (41). Hearing loss occurs in up to 85% of patients with MWS and is usually perceived in the second or third decade of life (42). Recently, Kuemmerle-Deschner et al. reported that 21 out of 23 patients with MWS perceived hearing loss, which were classified as a sensorineural hearing loss when the average hearing thresholds of 0.5, 1, 2, and 4 kHz were compared to age-matched normative data. An additional two patients were also classified as having a sensorineural hearing loss when the average hearing thresholds of 6 and 8 kHz were compared to normative data (43). These findings suggest that an early hearing loss in MWS affects high frequencies above 4 kHz and remains undetected by hearing assessment of middle frequencies essential for word recognition. Hearing assessment including high frequencies of 6 and 8 kHz may allow for the recognition of early hearing loss associated with MWS.

Additional auditory assessments, including speech recognition, loudness scaling, gap detection, and difference limen for frequency (DL_f_), were conducted in five affected family members from a Dutch family with MWS. Although speech recognition scores were clearly affected in these members, they had higher speech recognition scores than patients with presbyacusis with similar levels of hearing impairment, indicating that speech recognition was well-preserved in the MWS family. The loudness growth curves of the affected family members showed steeper slopes than those of individuals with normal hearing. Both sets of findings indicate that hearing loss was primarily of cochlear origin (41). In other patients with MWS, distortion product otoacoustic emissions (DPOAEs) were absent, consistent with a cochlear origin (42). The loudness growth curves in the MWS family are comparable to those of patients with intra-cochlear conductive hearing loss, such as DFNA8/12 (caused by *TECTA* gene variants) and DFNA13 (*COL11A2* gene), which are caused by tectorial membrane abnormalities. The mean gap detection result of the affected family members was close to that of individuals with normal hearing and was comparable to those of patients with DFNA8/12 and DFNA13. The mean DL_f_ of the present family members was poorer than that of individuals with normal hearing, but was fairly similar to those of patients with DFNA8/12 and DFNA13. Thus, the results of loudness scaling, gap detection, and DL_f_ of the affected family members were comparable to the results of patients with DFNA8/12 and DFNA13 with intra-cochlear conductive hearing loss, indicating that hearing loss in patients with MWS can be caused by the same pathogenesis (41).

Patients with NOMID usually show a progressive bilateral hearing loss. Hearing loss usually occurs within the first decade of life (44). In a prospective study, audiological data were collected from 38 patients with NOMID. Of these, 32 patients showed hearing loss and 4, 5, and 23 patients displayed conductive, mixed, and sensorineural hearing loss, respectively. Middle ear effusion (MEE) was present in nine patients, and a tympanostomy tube was present in three ears. MEE was much more prevalent than expected in patients with NOMID and is characteristic of conductive and mixed hearing loss in patients with NOMID. In contrast, MEE was found in none of the 12 patients with MWS. The authors speculated that proinflammatory cytokines such as IL-1β and TNF-α, known to regulate the expression of mucin and other genes involved in the pathogenesis of otitis media, and excessive IL-1β secretion in the respiratory mucosa, including the middle ear, may lead to more frequent otitis media in patients with NOMID (42). Thus, the hearing loss is mainly sensorineural in patients with NOMID, but MEE can also affect hearing, leading to conductive or mixed hearing loss.

Highly sensitive fluid-attenuated inversion recovery MRI (FLAIR-MRI) after the administration of gadolinium in the inner ear was performed to visualize potential inflammatory cochlear lesions (27). On FLAIR-MRI sequences, cochlear enhancement was reportedly found in 26 of 29 patients with NOMID, three of nine patients with MWS, and one of six patients with FCAS. Cochlear enhancement was more frequent in those with hearing loss than in those without hearing loss, suggesting that it can be an accurate predictor of hearing loss, when caused by cochlear inflammation (42).

### Auditory Response to Anti-IL-1 Therapy in Patients With CAPS

It should be noted that hearing loss in patients with CAPS can be improved or stabilized by inhibiting IL-1-mediated inflammation using anti-IL-1 drugs, such as anakinra and canakinumab. This is a unique characteristic of hearing loss in patients with CAPS, since it is usually difficult to treat genetic hearing loss by drugs (6). To date, a number of cohort studies have revealed varied results with treatment using anti-IL-1 drugs for patients with MWS and NOMID. In a cohort study of 23 patients with MWS aged from 3 to 72 years with a median age of 16 years, all patients showed at least a mild sensorineural hearing loss at high frequencies. A total of 44 out of 46 ears showed an improvement or stable hearing with anti- IL-1 drugs. Improvement was found in 11 ears, of which six were treated with canakinumab and five with anakinra. Stable hearing was found in 33 ears, of which 20 were treated with canakinumab and 13 with anakinra. Two ears worsened, both of which were treated with anakinra (43). In another cohort of 33 patients with MWS aged 3–75 years with a median age of 35 years, 22 of 33 patients had sensorineural hearing loss. Hearing was stable in the majority of patients treated with anti-IL-1 drugs. Hearing improved in five patients, of which three were treated with canakinumab and two with anakinra. Hearing deteriorated in one patient treated with anakinra (40). Thus, hearing loss can be improved or stabilized in the majority of patients with MWS by anti-IL-1 drugs. Younger patients were most likely to respond to treatment in both studies, indicating that there is a window of opportunity whereby response to treatment can be seen. A recent report including three patients with MWS is also consistent with the observation. Three affected family members, including a 2-year-old boy, the 34-year-old father, and the 65-year-old grandfather, were treated with canakinumab. Hearing thresholds examined by conditioned orientation response audiometry improved from 30–50 dB to 30 dB in the boy, while hearing did not improve in his father and grandfather (45).

Sibley et al. presented the data of 26 patients with NOMID aged 1–42 years with a mean age of 12 years. Eighteen of the 26 patients had at least a mild hearing loss in one ear. Hearing improvement was found in 30% of the cases, and progression of hearing loss was halted in the majority of the patients by treatment with anakinra. In this study, cochlear enhancement on gadolinium-enhanced FLAIR-MRI was evaluated and scored in patients with NOMID. Higher scores were observed for ears with progressive hearing loss compared to those with stable or improved hearing, indicating that cochlear enhancement scores were predictive of further hearing loss (46). In a cohort of 10 patients with NOMID aged from 3 months to 20 years, seven of 10 patients had hearing loss. Two younger patients exhibited a 15 dB-improvement 6 months after the start of anakinra therapy. The remaining five patients showed stable hearing after the dosage of anakinra had increased (47). It is notable here that persistent cochlear inflammation may damage the cochlear nerve and neuroepithelia to cause irreversible hearing loss in these patients, especially if there was a delay in diagnosis and treatment.

Recently, Kim et al. evaluated the audiological response to anakinra therapy in 13 patients with CAPS, including nine patients with NOMID, two with MWS, and two with FCAS. They analyzed the audiological response in relation to the initial hearing threshold, cochlear enhancement on gadolinium-enhanced FLAIR-MRI, and inflammatory markers. Patients with initial hearing thresholds worse than 60 dB did not recover better than hearing thresholds of 60 dB, though 18-year-old male with MSW recovered from severe to moderate severe hearing loss with anakinra therapy. In contrast, patients with initial hearing thresholds better than 40 dB did not get worse than hearing thresholds of 40 dB, except for one patient with NOMID. These findings indicate that there is a time window of opportunity to respond to treatment and that initial hearing thresholds worse than 60 dB suggests poor hearing prognosis. Cochlear enhancement on FLAIR-MRI was examined in nine patients. Of these patients, cochlear enhancement was found in two patients who presented initial hearing thresholds worse than 60 dB and progression of hearing loss in spite of anakinra therapy. Thus, cochlear enhancement was associated with poor hearing prognosis. Inflammatory markers, such as C-reactive protein (CRP) and erythrocyte sedimentation rate (ESR) were examined in all patients. The levels of CRP and ESR decreased immediately after anakinra therapy in most of the patients. However, sometimes the hearing deteriorated even when the inflammatory markers and systemic symptoms were well-controlled. Therefore, the dosage of anakinra required for controlling cochlear inflammation could be the highest among that required for the various tissues (48).

Thus, anti-IL-1 therapy can mitigate hearing loss in patients with MWS and NOMID. There is a window of opportunity to respond to treatment, and the younger patients were most likely to respond to treatment, probably because persistent cochlear inflammation may damage the cochlear nerve and neuroepithelia to make hearing loss irreversible. In brief, cochlear enhancement on gadolinium-enhanced FLAIR-MRI and initial hearing thresholds worse than 60 dB may indicate poor hearing prognosis. Anakinra dosage required for controlling cochlear inflammation may be higher than that required for yielding effective results in other organs.

### Auditory Performance After Cochlear Implant in Patients With CAPS

Audiologic performance was evaluated after CI in a patient with MWS and a patient with NOMID. Both patients had bilateral profound sensorineural hearing loss despite treatment with anakinra. The patient with MWS received CI in both ears at the age of 8 years, and the patient with NOMID received CI in the left ear at the age of 15 years. Their speech perception score, examined by the Korean version-central institute for the deaf (K-CID), reached 100% at 6 months post-CI, and was seemingly better than the CI outcomes of those with identified genetic etiology and those without identifiable genetic variants (49). These results suggest that CI is a viable and an effective treatment option for patients with MWS and NOMID. On CI operation, the cochlear round window was easily identified and electrodes were smoothly inserted into scala tympani without any resistance by ossification or granulation tissues in the patient with MWS and that with NOMID (48). These findings are different from those found in patients with non-*NLRP3* related autoinflammatory hearing loss. These patients occasionally show ossification around round window and granulation tissues in scala tympani of the basal turn. Cochlear inflammation via the activation of the NLRP3 inflammasome affects cochlear tissue differently from what is caused by non-*NLRP3* related autoinflammatory etiology.

### Vestibular Characteristics of Patients With CAPS

Detailed vestibular function was reported in three articles on patients with MWS. In a study by Kuemmerle-Deschner et al. the vestibular function was assessed in 15 patients with MWS by videonystagmography, including observation of spontaneous and head shake nystagmus and nystagmus after caloric stimulation. None of the patients complained of vertigo. Neither spontaneous nor head-shake nystagmus was observed. Caloric stimulation showed a normal nystagmus response in all the patients (40). In a cohort of 23 patients with MWS, vestibular function was assessed by observing nystagmus induced by caloric stimulation. All patients showed a normal nystagmus response to caloric stimulation (43). In a study by Weegerink et al., the vestibular function was evaluated in three of six patients with MWS by videonystagmography tests, including the evaluation of saccadic, smooth pursuit, and optokinetic nystagmus responses, and the observation of nystagmus after rotatory and caloric stimulation. The three patients had variable vestibular symptoms, such as dizziness and instability, especially in the dark. Evaluation of the vestibular function in the first patient revealed no abnormalities. In contrast, the second patient showed hyporeflexia of velocity-step responses and weak nystagmus responses after a caloric stimulation in both ears. The third patient showed hyporeflexia in the rotatory test, while caloric stimulation results revealed a normal response (41). Thus, the vestibular function in patients with MWS is variable, and more patients should be tested to assess whether the vestibular function is affected.

## DFNA34

### Overview of DFNA34

DFNA34 is an autosomal dominant non-syndromic sensorineural hearing loss caused by *NLRP3* variants (50). A genome-wide linkage analysis was performed in three generations of a North American Caucasian family (LMG113 family) segregating sensorineural hearing loss, and revealed a positive region on chromosome 1q43–1q44. The DFNA34 interval included 36 protein-coding genes and dideoxy sequence analysis of the genes identified a heterozygous variant c.2753G>A (p.Arg918Gln) in the *NLRP3* gene. To evaluate the pathogenicity of the variant, peripheral blood monocytes from three affected individuals were cultured with lipopolysaccharide and the cells secreted high levels of IL-1β compared with non-detectable levels of IL-1β secreted by healthy control monocytes. Postcontrast FLAIR-MRI examination revealed cochlear enhancement in two affected members, indicating cochlear inflammation (50).

### Hearing Characteristics of Patients With DFNA34

All eight affected individuals from the LMG113 family had bilateral, symmetric sensorineural hearing loss with a reported onset in the late second to fourth decade of life. Their hearing loss was slowly progressive and initially affected at high frequencies. Low and middle frequencies were affected by advancing age, resulting in a moderate hearing loss with a downsloping audiometric configuration. DPOAEs were absent in a patient examined. The average annual threshold deterioration was 0.9–1.5 dB/year with high values in high frequencies. Speech discrimination scores ranged from 60 to 100% (51). Absence of DPOAEs and the good speech discrimination scores indicate that hearing loss was of cochlear origin, but not with retrocochlear etiology. One patient was treated with anakinra for 110 days, but the mean hearing thresholds after treatment were within 5 dB compared with those before treatment. None of the patients complained of vertigo (51).

The second case of DFNA34 was reported by Kim et al. The proband was a 34-year-old woman with bilateral severe sensorineural hearing loss without any syndromic features characteristic of CAPS. Mutation analysis revealed c.2752C>T (p.Arg918X) variant in the *NLRP3* gene. Her 18-month-old daughter showed bilateral mild hearing loss and had the same variant, consistent with autosomal dominant inheritance. The proband was reluctant to undergo anakinra therapy and received CI at the age of 34 years. Her speech perception score examined by K-CID reached 100% at 6 months post-CI, suggesting that CI is a viable and effective treatment option for patients with DFNA34 (48, 49).

Thus, two families from North American Caucasian and Korean populations were reported to have DFNA34. Surprisingly, both families have variants affecting the same amino acid, Arg918, located in the LRR domain in NLRP3. These results indicate that variants affecting Arg918 cause non-syndromic sensorineural hearing loss without inflammatory signs or symptoms in any other organ.

## Etiology of Hearing Loss in Patients With CAPS and DFNA34

Although the mechanism of hearing loss in CAPS and DFNA34 is still under research, some data had shed light on its etiology. Well-preserved speech recognition scores and absence of DPOAEs in patients with MWS and DFNA34, as mentioned above, are consistent with hearing loss being of cochlear origin, but not of retrocochlear etiology (41, 51). Cochlear enhancement on postcontrast FLAIR-MRI was found in patients with NOMID, MWS, and DFNA34, indicating cochlear inflammation and its association with hearing loss (42, 50). Though the mechanism underlying cochlear inflammation remains unclear, cochlear inflammation can be induced through one or both of the following mechanisms. Systemic autoinflammation induces cochlear inflammation as a secondary target organ or local inflammatory cells induce cochlear inflammation. Ahmadi et al. speculated that NLRP3 inflammasome activation causes an inappropriate secretion of IL-1β, leading to chronic aseptic meningitis and subsequently, increased permeability of cytokines between the perilymph and cerebrospinal fluid space via the modiolus. These cytokines can then stimulate spiral ligament fibrocytes to produce downstream mediators that may induce cochlear inflammation responsible for cochlear dysfunction and hearing loss (42). However, their speculation is not consistent with the findings that patients without chronic aseptic meningitis show sensorineural hearing loss (40, 43). In contrast, we hypothesized that resident macrophage/monocyte-like cells in the cochlea can mediate local autoinflammation via the activation of the NLRP3 inflammasome, leading to cochlear dysfunction in patients with DFNA34, since hearing loss segregates without any other target organ manifestations. We demonstrated that macrophage/monocyte-like cells were scattered throughout mouse cochlear tissues in which the NLRP3 inflammasome could be activated with secretion of IL-1β. Based on these findings, we concluded that local cochlear activation of the NLRP3 inflammasome could induce cochlear autoinflammation and sensorineural hearing loss (50).

Muckle and Wells reported the findings of postmortem examinations of the temporal bones of two patients with MWS in 1962. Both patients had shown progressive hearing loss since childhood. Postmortem examinations revealed degeneration of the cochlear nerve, the organ of Corti, and vestibular sensory epithelium (52). In contrast, the results of loudness scaling, gap detection, and DL_f_ in patients with MWS were comparable to the results of patients with DFNA8/12 and DFNA13 with intra-cochlear conductive hearing loss, indicating that hearing loss in patients with MWS can be caused by the same pathogenesis (41). Postmortem examination of the human temporal bone enables assessing the etiology of hearing loss. However, human temporal bones are collected only in a few laboratories. In addition, they are affected by postmortem autolysis. It is difficult to predict specific cellular damage, such as inner/outer hair cell loss, degeneration of spiral ganglion neurons, and atrophy of stria vascularis, only based on subjective hearing assessments, such as audiometry and speech discrimination test (53). Comprehensive hearing assessment including DPOAE and auditory brainstem response, in addition to that using the newly emerging non-/minimally-invasive tools, such as cochlear endoscopy, is required to elucidate the etiology of cochlear dysfunction and the mechanism underlying cochlear inflammation.

## Conclusion

Patients with CAPS and DFNA34 show progressive bilateral sensorineural hearing loss. Hearing loss has unique characteristics that can be improved or stabilized by anti-IL-1 therapy. However, it should be noted that there is a window of opportunity to respond to treatment, and younger patients are most likely to respond. It is important to know the characteristics of CAPS and DFNA34 for early diagnosis, and mutation analysis of *NLRP3* will lead to a definite diagnosis. Although the etiology of hearing loss is not fully understood, the mechanism may be a final common pathway for hearing loss caused by a variety of factors that can activate innate immunity, such as pathogens, chemicals, trauma, aging, or oxidative stress. Future investigations into the role of NLRP3 in the cochlea and the pathogenesis of hearing loss may shed light on the etiology of other sensorineural hearing loss cases.

## Author Contributions

HN conceived the study. All authors wrote the manuscript, reviewed its drafts, approved its final version, and agreed with its submission.

## Funding

This study was funded by a Health and Labor Sciences Research Grant for Research on Rare and Intractable Diseases and Comprehensive Research on Disability Health and Welfare from the Ministry of Health, Labor and Welfare of Japan (HN 20FC1048) and JSPS KAKENHI Grant Number JP 20K09729 (HN).

## Conflict of Interest

The authors declare that the research was conducted in the absence of any commercial or financial relationships that could be construed as a potential conflict of interest. The reviewer MH is currently organizing a Research Topic with the author HN.

## Publisher's Note

All claims expressed in this article are solely those of the authors and do not necessarily represent those of their affiliated organizations, or those of the publisher, the editors and the reviewers. Any product that may be evaluated in this article, or claim that may be made by its manufacturer, is not guaranteed or endorsed by the publisher.
